# The Relationship between Internet Addiction, Internet Gaming and Anxiety among Medical Students in a Malaysian Public University during COVID-19 Pandemic

**DOI:** 10.3390/ijerph182211870

**Published:** 2021-11-12

**Authors:** Nurazah Ismail, Ahmad Izzat Tajjudin, Hafiz Jaafar, Nik Ruzyanei Nik Jaafar, Azlin Baharudin, Normala Ibrahim

**Affiliations:** 1 Psychiatry Unit, Department of Medicine, Faculty of Medicine and Health Sciences, Universiti Sains Islam Malaysia, Nilai 71800, Malaysia; azah.ismail@usim.edu.my (N.I.); izzattajuddin@usim.edu.my (A.I.T.); 2 Malaysian Society of Internet Addiction Prevention, Universiti Putra Malaysia (UPM), Serdang 43400, Malaysia; ruzyanei@ppukm.ukm.edu.my (N.R.N.J.); normala_ib@upm.edu.my (N.I.); 3 Public Health Unit, Department of Primary Health Care, Faculty of Medicine and Health Sciences, Universiti Sains Islam Malaysia, Nilai 71800, Malaysia; dr.hafizjaafar@usim.edu.my; 4 Department of Psychiatry, Faculty of Medicine, Universiti Kebangsaan Malaysia Medical Centre, Jalan Yaacob Latif, Kuala Lumpur 56000, Malaysia; 5 Department of Psychiatry, Faculty of Medicine and Health Sciences, Universiti Putra Malaysia (UPM), Serdang 43400, Malaysia

**Keywords:** internet addiction, internet gaming, medical students, anxiety, COVID-19

## Abstract

The internet has become an important medium for learning and communication during the COVID-19 pandemic, particularly for university students. Nevertheless, an increase in internet usage could predispose people to internet addiction (IA) and internet gaming (IG). Equally, there is concern that anxiety levels have increased during the pandemic. The aim of this study is to determine the prevalence of IA and IG, and their associations with anxiety among medical students during the pandemic. Data were collected during the second wave of the “Conditional Movement Control Order” (CMCO) in Malaysia between 12 November and 10 December 2020. A total of 237 students participated through proportionate stratified random sampling in this cross-sectional study. They completed a set of online questionnaires which consisted of a sociodemographic profile, the Malay version of the internet addiction test (MVIAT), the Malay version of the internet gaming disorder-short form (IGDS9-SF) and the Malay version of the depression, anxiety and stress scale (DASS-21). The prevalence of IA and internet gaming disorder (IGD) were 83.5% and 2.5%, respectively. A multiple logistic regression showed that those in pre-clinical years had a greater risk of anxiety than those in clinical years [(AOR) = 2.49, *p*-value 0.01, 95% CI = 1.22–5.07]. In contrast, those who scored high on IA were protected against anxiety [AOR = 0.100, *p*-value 0.03, 95% CI = 0.01–0.76)]. In conclusion, IA was highly prevalent during the COVID-19 pandemic and its high usage might serve as a protective factor against anxiety among the medical students in this study sample.

## 1. Introduction

The advent of a deadly virus, coronavirus virus disease 2019 (COVID-19), has had a profound impact on everyone. As a result of COVID-19’s large-scale outbreak infection, the World Health Organization (WHO) declared the outbreak as a pandemic in March 2020 and required rapid and extensive measures to keep from the spreading of the disease. As soon as the disease transmission increased in March 2020, it prompted the government to implement a “Movement Control Order” (MCO) to curb the spreading of the virus. This was imposed in phases until it entered a recovery phase beginning on the 10th of June onwards [1]. Unfortunately, the country experienced a second wave of COVID-19 in October 2020, which led to the enforcement of the Conditional Movement Control Order (CMCO) with a tighter standard operation procedure (SOP) than the earlier recovery phase. The CMCO was extended until early December 2020 as Malaysia continued its fight against the pathogen and certain economic activities were allowed to function under strict SOPs [2].

During the COVID-19 pandemic, the internet has taken a more prominent role and became the most common means of learning, communication, and conveying information. This was also reflected by a high number of internet users in Malaysia as reported in the Internet Users Survey (IUS) by the Malaysian Communication and Multimedia Commission (MCMC) in 2020, where 88.7% were internet users [3]. Students, particularly, were affected by the switch from the conventional face-to-face learning to virtual platforms. This could predispose them to internet-related problems such as internet addiction (IA) and internet gaming disorder (IGD) [4].

In a meta-analysis study from 31 countries, the global prevalence of IA before the pandemic was found to be around 6.0% [95% CI 5.1–6.9], with the highest prevalence found in the Middle East at 10.9% [95% CI 5.4–6.3] and the lowest prevalence found in Northern and Western Europe at 2.6% [95% CI 1.0–4.1]. The prevalence of IA in Asian countries was high at 7.1% [5]. Among medical students, another meta-analysis showed that the pooled prevalence of IA pre-pandemic was 30.1% [95% CI 28.5–31.8%], whereby the prevalence was 36.9% for Malaysia [6,7,8]. When compared between the global prevalence and studies conducted among medical students, the prevalence rate was six times higher than that of the general population, placing them among the vulnerable group.

For IG, it can be a temporary method for stress relief. However, it may become a disorder when the coping turns out to be maladaptive and subsequently leads to disruptions in major areas of functioning. The prevalence of internet gaming disorder (IGD) in European countries pre-pandemic varied from 1.2% to 5.0%, compared to a higher range of prevalence among Asian countries, i.e., from 7.5% to 26.7% [9]. The local prevalence for IG is 18% [10]. A recent study on IGD among Malaysian undergraduates found that 52.8% of them scored high on a validated IGD scale [11]. The disparity across these figures in IA and IGD may be attributed to the use of different objective assessment tools and the heterogeneity of the sample population [12].

This pandemic has left significant psychological impacts such as insomnia, anxiety and depression [13,14,15] in addition to the direct effects of the virus itself [16]. Anxiety has been highly prevalent and affects various subpopulations differently depending on their risk profile [17,18]. The constant and ongoing stressors for medical students placed them among the vulnerable group attributed to social isolation, suspension of studies, impact of online learning and possibly limited clinical exposure [19].

A recent study conducted among Malaysian youth during the pandemic which examined the relationship between anxiety and IA, found that individuals with higher internet usage scored more in the anxiety inventory [20]. Therefore, this study aimed to determine the prevalence of internet usage and IG among medical students and whether they are linked to anxiety during the pandemic. Understanding these factors would provide insight for the university management and other stakeholders on adaptive strategies during pandemic times to facilitate medical students’ learning and optimize care for their psychological wellbeing.

## 2. Materials and Methods

**Figure 1:**
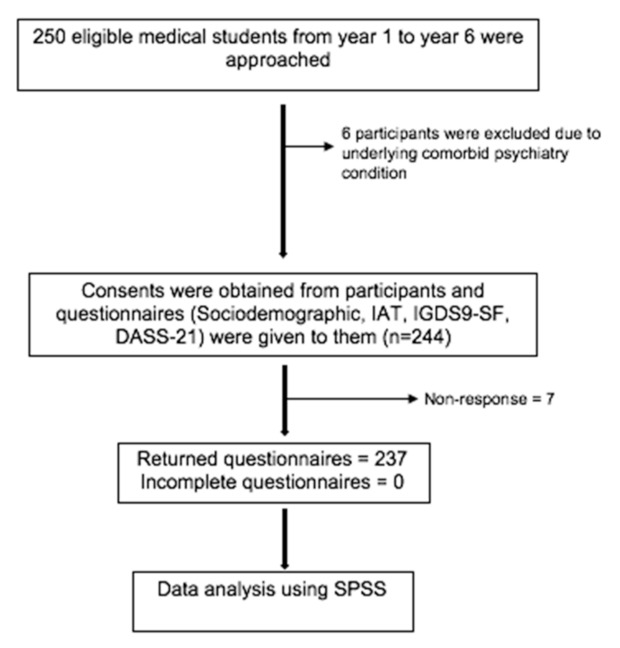
Flow chart of data collection.

This is a cross-sectional study conducted among medical students in a Malaysian public university. The survey was opened to all medical students in the Islamic Science University of Malaysia (USIM) who had been using the internet for the past 12 months or more. Those who identified themselves as currently under psychiatric follow-up and/or in treatment were excluded from the study. A total of 250 students (from all academic years) were recruited through proportionate stratified random sampling as described in Figure 1 [21]. The randomization was generated using a computer software. Those who consented to participate in the study were given a set of self-rated, pre-tested and validated questionnaires for internet addiction, internet gaming and anxiety using Google Form. This was due to the restricted movement during the COVID-19 pandemic in Malaysia. Respondents were required to fill out the sociodemographic questionnaire consisting of their information regarding age, gender, marital status, ethnicity, internet gaming characteristics and internet use pattern. The Malay version of the internet addiction test (MVIAT) is a self-reported questionnaire to measure the characteristic and behaviour patterns of internet usage including the functionality in major important areas [22]. The sensitivity and specificity were 63% and 61%, respectively in the validated MVIAT. The Cronbach alpha was 0.91 [23,24]. The Malay version of the internet gaming disorder scale-short form (IGDS9-SF) was used to measure internet gaming behaviour and its severity [25]. The sensitivity and specificity were 98.0% and 91.9%, respectively, in the validated Malay version [26,27]. The Cronbach alpha was 0.87 in the present study [28]. The Malay version of the depression, anxiety, stress scale-21 (DASS-21) was used as a screening tool for anxiety level in this study. The Cronbach alpha in the validated Malay version was 0.74 for anxiety [29]. Data collection was conducted during the second wave of the CMCO period between 12 November 2020 and 10 December 2020.

## 3. Data Analysis

A descriptive analysis was conducted to determine the prevalence of IA and IGD as well as the sociodemographic characteristics of the study population, academic backgrounds and the internet use characteristics. The results were presented as frequency and percentage for categorical data and mean (standard deviation) for continuous data. A bivariate analysis was performed to establish the association between the following factors: gender, marital status, hometown, household income, internet use characteristics and academic years with (1) MVIAT and (2) Malay version of the IGDS9-SF. The level of significance was pre-set at 0.05. The completed MVIAT and the Malay version of the IGDS9-SF were scored according to manual guidelines and literature reviews and all the scores were categorized into dichotomous groups. The bivariate analysis was also conducted between all the independent variables with the anxiety component in DASS-21 as the outcome. DASS-21 scores were categorized into dichotomous groups for a score of more than 7 for anxiety based on the descriptive cut-off score provided by the creator of the scale [29]. A bivariate analysis using Pearson Chi-square and Fisher’s exact test was conducted to determine the association between the independent variables and the dependent variable. Significant bivariate analysis results were further analysed using a multiple logistic regression analysis. The collected data was analysed using the IBM SPSS (New York, NY, USA) statistical software program version 26.

## 4. Results

The summaries of the participants’ sociodemographic characteristics are described in Table 1. The male participants represented 30.4% of the population. The age of the participants was not normally distributed where the z-value was 0.315 (skewness 0.143/SE 0.158) and Kolmogorov–Smirnov significant level was <0.05 (*p* ≤ 0.001). The median age was 21.00 (3.0).

Table 2 shows the association between sociodemographic characteristics, academic background, and internet use characteristics with anxiety. Among all of these, only academic years showed a significant association with anxiety whereby a greater number of those in pre-clinical years had anxiety compared to those in clinical years (χ^2^ = 6.739; df = 1; *p* = 0.013). A Chi-square analysis revealed that IA was not significantly associated with IGD (*p* > 0.05).

Table 3 shows the multiple logistic regression between the independent variables with anxiety. The model contained independent variables from which all the factors were statistically significant, χ^2^ (2, *n* = 237) = 18.62, *p* < 0.001, indicating that the model was able to differentiate between the respondents who reported and did not report being anxious. The strongest factor reported related to having anxiety was being in the pre-clinical years. The odds of having anxiety is 2.5 times more likely among pre-clinical years compared to clinical years, controlling for the other factors in this model [adjusted odds ratio (AOR) = 2.489, *p*-value 0.01, 95% CI = 1.22–5.07]. However, those with a greater IAT score had a lower anxiety level by 0.10 (AOR = 0.100, *p*-value 0.03, 95% CI = 0.01–0.76), controlling for other factors in this model.

## 5. Discussion

This study revealed a drastic increase in the prevalence of IA, i.e., 83.5% among medical students during the pandemic compared to a pre-pandemic study, which reported its highest prevalence at 37% [6]. Studies on IA across the world during the pandemic era are limited. Furthermore, there is a wide variation of its prevalence among countries. A recent study conducted in Indonesia revealed the prevalence of IA among adults was only 15%, while in Nigeria, the prevalence was up to 55% among university students [30,31]. In another study, among the general population in Taiwan, the prevalence of IA was only 24.4% which was lower than the prevalence of this study [32,33]. The high prevalence of IA among medical students could be attributed to the COVID-19 pandemic itself. The current pandemic situation has prompted most people to spend more time in front of the computers or smartphones, maximizing their internet functions and benefits. This is particularly the case for students, as they were required to switch from the conventional learning environment to virtual platforms. In most cases, the switch had been swift and smooth since Malaysia’s internet connectivity and accessibility are generally good [3].

The use of the internet via smartphone or computer has certainly increased during the pandemic. Because of the lack of physical outdoor activities in keeping with COVID-19 restrictions, it has been utilised for a wide array of purposes besides being used for education, such as holding virtual workouts, doing online shopping and virtual socializing. Interactions with family members and friends were made safer and more convenient on the internet, and this served the purpose of complying with the restricted movement order to flatten the number of COVID-19 new cases [34]. However, the long-term effect of a prolonged and more frequent usage of the internet could predispose vulnerable individuals to IA [35].

For internet gaming, a meta-analysis study reported that the worldwide prevalence of IGD before the pandemic was 3.05% [36]. A different meta-analysis study conducted across Southeast Asian countries revealed the pre-pandemic prevalence of IGD varied from 5.4% to 15.4% [37]. There is limited information on IGD locally during the pandemic period. A European study performed among the adult population during the pandemic showed that the prevalence of IGD was 29.6% [38], while a study in Japan discovered that there was 1.6 times increment of the prevalence, from 3.7% to 5.6% before and during pandemic [39]. In this study, we found that the prevalence of IGD was only 2.5%, which is the lowest among other countries [9,10,36,39]. The discrepancies in the global prevalence could be the result of the non-uniform instrument tool used, the current pandemic situation, and different population samples [36,40]. A common explanation for the relatively high rate of IGD during this pandemic period is that internet gaming serves as a stress reliever to cope with pandemic anxiety and stress [41,42,43,44]. Nevertheless, our study did not find a high prevalence of IGD nor any significant association with anxiety among the medical students during the pandemic.

The prevalence of anxiety among the medical students in this study was 19.4%. This finding was lower than those found in other studies worldwide, i.e., between 30–50%, but comparable to the local prevalence of 22 to 30% [45,46,47]. This study found that those in pre-clinical years had a 2.5 times greater likelihood of developing anxiety than those in clinical years. Multiple factors played a role in inducing anxiety in both the pre-clinical and clinical years of a medical education. It may be due to the environmental factors (transitional phase from secondary school to tertiary education, adjustment to a new life, competitive environment), academic factors (excessive educational content, multiple examinations, expectation to master the knowledge) and personal factors (individual personality, parental expectation, fear of inadequacy) [47,48,49]. One plausible explanation is that those in the clinical years were still allowed to attend clinical sessions during CMCO; therefore, they experienced reduced social isolation which in turn lowered their anxiety level compared to those in pre-clinical years who had to have only online classes.

In stark contrast to the earlier pre-pandemic study findings that showed IA was linked to anxiety and other psychological impacts [46,50], this study reported those who scored as internet dependent were protected against anxiety. It is probable that excessive internet use served as a self-therapeutic method to escape from academic stress, and pandemic burn-out by reducing the feeling of social isolation, overcoming loneliness, having easy access to information and perhaps even providing a suitable medium for those with social anxiety to improve their social skills [51,52,53]. A further exploration regarding the motivation and purpose of the internet would be useful when examining the internet use among medical students to be more conclusive of how the internet could either be beneficial or harmful during stressful times such as the viral pandemic.

For internet gaming, other studies showed that IGD posed a significant association with psychological distress [54,55]. Even though internet gaming offers an easy way to escape from everyday life and potentially serves as a stress coping skill for negative emotions, there are definite factors that may protect people from being involved with too much internet gaming, in contrast to the internet use as shown in this study. These include the sociodemographic background of the students and individual factors (preference, personality), cognitive factors (IQ and perseverative errors), psychopathological conditions (underlying depression, anxiety and impulsivity), social interaction factors (family environment, social anxiety and self-esteem) as well as the nature of the study program itself [56,57,58,59].

Though the need for the usage of information and communication technology (ICT) is crucial during this difficult time and we could develop a dependence on it, its inherent risk can also be outweighed by its potential benefits. Nevertheless, caution must be undertaken for those who are vulnerable to developing overdependence [60]. Yet, at this point of time, it is difficult to limit the use of ICT. Therefore, it is very important for the university management to be able to detect problematic usage early to avoid potential future problems [61]. Preventive measures for both physical and mental health include: self-regulating screen time by limiting and balancing screen and internet time for important matters (education and searching information), preventing aimless browsing of the internet, establishing routine by turning off notifications during physical activities, enhancing and empowering physical activities either indoors or outdoors such as forest-bathing, strolling and jungle trekking (once permitted), managing stress sufficiently through a relaxation technique or spiritual technique, regular and adequate sleep, healthy eating and seeking help when needed [62,63,64,65].

## 6. Conclusions

In conclusion, this study showed that the prevalence of internet addiction during the pandemic was higher than the rates found during the pre-pandemic period. Nevertheless, the prevalence of internet gaming disorders remained as low as the rates reported prior to the pandemic. Medical students in the pre-clinical academic years were at a greater risk of anxiety while those who rated higher on the internet addiction scale were protected against anxiety.

## 7. Limitation

This study had several limitations. Firstly, it used a cross-sectional study design whereby causal relationships could not be established. Secondly, the Malay version of IGDS9-SF used in this study had no locally validated cut-off point that would help to determine the proportion of those who needed a further clinical assessment for IGD. Lastly, the study population was homogenous from one university; all of whom were Malay Muslims and therefore would not represent all medical students in Malaysia. Nevertheless, its main strength is that, to the authors’ knowledge, it is the first study to explore the relationship between internet use, online gaming and psychological wellbeing of medical students in consideration of the prominent use of the internet during the COVID-19 pandemic.

## Figures and Tables

**Table 1:** Sociodemographic Profiles of Participants (*n* = 237).

Characteristics	*n*	%	Median (IQR)
Gender			
Male	72	30.4	
Female	165	69.6	
Age in years			21.00 (3.0)
19	37	15.6	
20	46	19.4	
21	37	15.6	
22	35	14.8	
23	45	19.0	
24	34	14.3	
25	2	0.8	
26	0	0	
27	1	0.4	
Religion			
Muslim	237	100	
Ethnicity			
Malay	235	99.2	
Chinese	0	0	
Indian	1	0.4	
Others	1	0.4	
Marital status			
Single	234	98.7	
Married	3	1.3	
Divorced	0	0	
Hometown			
Rural	96	40.5	
Urban	141	59.5	
Parental household income			
<RM 2000	34	14.3	
>RM 2000	203	85.7	
Academic years			
Pre-clinical	119	50.2	
Clinical	118	49.8	
Supplementary exam			
Yes	9	3.8	
No	228	96.2	
Total screen time (hours/day)			
<7 h	46	19.4	
>7 h	191	80.6	
Type of gadget			
Computer	18	7.6	
Laptop	218	92.0	
Smartphone	236	99.6	
Tablets	100	42.1	
Video game console	21	8.9	
Ownership of gadget			
Personally owned	232	97.9	
Shared with others	5	2.1	
Internet accessibility			
At home	237	100	
Library	32	13.5	
Cybercafé	7	3.0	
Hostel	140	59.1	
Faculty	125	52.7	
Public areas	71	30.0	
Purpose(s) of using internet			
Education	57	24.1	
Social networking	228	96.2	
Online gaming	89	37.6	
Internet chatting	234	98.7	
Online shopping	187	78.9	
Blogs	25	10.5	
Sexual activities	3	1.3	
Surfing for leisure	144	60.8	
Email	160	67.5	
Social media ownership			
Facebook	200	84.4	
Twitter	147	62.0	
Instagram	217	91.6	
WhatsApp	234	98.7	
YouTube	190	80.2	
WeChat	6	2.5	
Others	56	23.6	
Video game genre			
Not playing video game	95	41.4	
MMORPG	57	24.1	
Action/adventure	35	14.8	
First-person shooter	46	19.4	
Sports	24	10.1	
Rhythm	18	7.6	
Driving	25	10.5	
Real time strategy	48	10.2	
Puzzle	58	24.5	
Board & card games	38	16.0	
Gambling	5	2.1	

Abbreviations: RM, Malaysian Ringgit; MMORPG, Massively Multiplayer Online Role-Playing Game.

**Table 2:** Association Between Sociodemographic Characteristics, Academic Background And Internet Use Characteristics With Anxiety (*n* = 237).

Variables	Anxiety		Test Statistics		
	No *n* (%)	Yes *n* (%)	χ^2^	df	*p*-Value
Gender			0.497	1	0.593
Male	60 (83.3)	12 (16.7)			
Female	131 (79.4)	34 (20.6)			
Marital Status			0.377 ^a^	1	0.478
Single	189 (80.8)	45 (19.2)			
Married	2 (66.7)	1 (33.3)			
Hometown			0.298	1	0.620
Urban	112 (79.4)	29 (20.6)			
Rural	79 (82.3)	17 (17.7)			
Parental household income			2.844 ^a^	1	0.105
<RM 2000	31 (91.2)	3 (8.8)			
>RM 2000	160 (78.8)	43 (21.2))			
Academic years			6.739	1	0.013 *
Pre-clinical	88 (73.9)	31 (26.1)			
Clinical	103 (87.3)	15 (12.7)			
Supplementary exam			0.047 ^a^	1	0.688
Yes	7 (77.8)	2 (22.2)			
No	184 (80.7)	44 (19.3)			
Ownership of gadget			0.001 ^a^	1	1.000
Personally owned	187 (80.6)	45 (19.4)			
Shared with others	4 (80.0)	1 (20.0)			
Total screen time/day			1.479	1	0.300
<7 h	40 (87.0)	6 (13.0)			
>7 h	151 (79.1)	40 (20.9)			

Note: ^a^ Fisher’s exact test, * Significant at *p* < 0.05; Abbreviation: RM, Malaysian Ringgit.

**Table 3:** Multiple Logistic Regression Between Sociodemographic Characteristics, Academic Background And Internet Use Characteristics With Anxiety Among Medical Students.

Factors	AOR	95% CI	*p*-Value
Gender			
Male	1		
Female	0.775	0.35–1.71	0.527
Marital		0.02–8.72	0.587
Single	0.436		
Married	1		
Hometown			
Urban	1		
Rural	1.08	0.53–2.19	0.830
Parental income			
<RM 2000	1		
>RM 2000	0.479	0.14–1.70	0.256
Academic years			
Pre-clinical	2.489	1.22–5.07	0.012 *
Clinical	1		
Supplementary exam			
Yes	1.422	0.23–8.72	0.704
No	1		
Total screen time/day			
<7 h	1		
>7 h	0.553	0.21–1.44	0.225
Ownership of the gadget			
Personally owned	0.771	0.07–8.07	0.828
Shared	1		
Internet dependence			
Normal	1		
IAT	0.100	0.01–0.76	0.026 *
IGDS9-SF			
Non-Disorder	1		
Disordered	0.240	0.04–1.33	0.103

Note: * Significant at *p* < 0.05, reference = 1, Abbreviations: RM, Malaysian Ringgit; IGDS9-SF, Internet Gaming Disorder Scale-Short Form.

## Data Availability

All the data are based on the current study analysis. The data presented in this study are available on request from the corresponding author.
